# Sleep duration, sleep quality, and daytime napping in relation to incident cardiometabolic multimorbidity across three national aging cohorts: a prospective, interpretable machine learning study

**DOI:** 10.3389/fnut.2026.1850552

**Published:** 2026-07-30

**Authors:** Fanchang Wang, Hongyang Qiao, Yi Zheng, Shuang Wu, Yuxin Ni, Xiaoming He

**Affiliations:** 1 The Second School of Clinical Medicine, Zhejiang Chinese Medical University, Hangzhou, China; 2 The First Affiliated Hospital of Wenzhou Medical University, Wenzhou, China.; 3 Department of Clinical Nutrition, The Second Affiliated Hospital of Zhejiang Chinese Medical University, Hangzhou, China; 4 Department of Endocrinology and Metabolism, The Second Affiliated Hospital of Zhejiang Chinese Medical University, Hangzhou, China

**Keywords:** aging cohorts, cardiometabolic multimorbidity, interpretable machine learning, nighttime sleep duration, sleep quality

## Abstract

**Background:**

Sleep disturbances are modifiable factors linked to metabolic dysregulation, adiposity, blood pressure abnormalities, and cardiometabolic risk. Evidence remains limited on whether nighttime sleep duration, sleep quality, and daytime napping predict cardiometabolic multimorbidity (CMM), defined as two or more of hypertension, type 2 diabetes mellitus, coronary heart disease, and stroke, across populations.

**Methods:**

Using CHARLS, ELSA, and HRS, we examined associations between sleep patterns and incident CMM in prospective aging cohorts. Kaplan–Meier curves and Cox proportional hazards models were used for time-to-event analyses, adjusting for sociodemographic characteristics, physical activity, and clinical covariates. Restricted cubic splines explored nonlinear associations. Mediation analyses assessed whether blood pressure, lipid markers, and C-reactive protein mediated the association between nighttime sleep duration and CMM. Interpretable machine learning evaluated the contribution of sleep variables within the cardiometabolic risk profile. Because sleep measures differed across cohorts, harmonization was conducted at the construct rather than raw-score level, and cross-cohort effect-size comparisons should be interpreted cautiously.

**Results:**

The analysis included 13,859 participants: 4,145 from CHARLS, 4,268 from ELSA, and 5,446 from HRS. In fully adjusted Cox models, good sleep quality was associated with lower CMM risk than poor sleep quality in CHARLS and ELSA. A similar trend was observed in HRS, although significance appeared only in complete-case sensitivity analysis. Kaplan–Meier curves showed poorer CMM-free survival among participants with poor sleep quality. For nighttime sleep duration, restricted cubic splines suggested a U-shaped association in ELSA before adjustment, but this weakened after full adjustment, and findings were inconsistent across cohorts. In exploratory mediation analyses, some small indirect effects were observed in unadjusted models, but none remained evident after covariate adjustment. In prediction analyses, logistic regression and LightGBM showed stable performance. SHAP identified systolic blood pressure, waist circumference, and BMI as leading predictors, while nighttime sleep duration added predictive information.

**Conclusion:**

Better sleep quality was associated with lower incident CMM risk in CHARLS and ELSA, with a similar but less robust pattern in HRS. Nighttime sleep duration showed less consistent associations. Machine learning analyses were exploratory and supported risk stratification and feature interpretation rather than clinical screening.

## 1 Introduction

Cardiometabolic multimorbidity (CMM) refers to the co-occurrence of two or more of the following conditions: hypertension, type 2 diabetes mellitus, coronary heart disease, and stroke (1). This complex chronic condition is a major burden on adults worldwide. It leads to higher mortality, disability, and healthcare use (2–5). This is especially true in large, diverse countries such as China, the United Kingdom, and the United States (6, 7). Identifying modifiable early risk factors and developing scalable prevention strategies are important for delaying the onset and progression of CMM.

Sleep disturbances are getting more attention in cardiometabolic research. They are common and can be changed. In addition to known links with hypertension and diabetes, disrupted sleep is also linked to adiposity, insulin resistance, and dyslipidemia (8, 9). Each adds to the cumulative cardiometabolic burden. At the mechanistic level, insufficient or excessive nighttime sleep, poor sleep quality, insomnia symptoms, and circadian misalignment may contribute to metabolic and vascular problems (10–12). This occurs through several pathways: neuroendocrine activation, sympathetic overactivity, chronic low-grade inflammation, oxidative stress, and changes in appetite- and energy-related hormones such as leptin and ghrelin (13–17). Despite these mechanisms, it remains unclear whether nighttime sleep duration, sleep quality, and daytime napping each independently and consistently predict CMM, especially across different sociocultural settings. A main limitation of existing research is its focus on single sleep measures, specific outcomes, or composite indicators. This makes it hard to study the range of sleep patterns and their possible links with CMM.

At the methodological level, machine learning provides a distinct advantage for disentangling the complex associations between sleep and CMM. Unlike conventional approaches, machine learning can capture nonlinear relationships and higher-order interactions across high-dimensional feature spaces (18, 19), making it particularly well suited to modeling risk structures arising from the interplay among multiple sleep dimensions, lifestyle factors, and sociodemographic variables. Adding interpretability methods may help explain the role of sleep-related variables in the model and improve the practical value of the findings. These methods may also help identify sleep patterns and participant groups that could benefit most from targeted prevention.

Using data from national aging cohorts in China, the United Kingdom, and the United States, we examined whether nighttime sleep duration, sleep quality, and daytime napping were associated with incident CMM. Daytime napping was available only in CHARLS. We further used interpretable machine learning to assess the contribution of sleep variables together with other cardiometabolic factors. Comparisons across cohorts were used to explore whether these associations were consistent across populations.

## 2 Methods

### 2.1 Study design and participants

Three population-based prospective aging cohorts were included: CHARLS in China, ELSA in the United Kingdom, and HRS in the United States (20–22). Baseline corresponded to Wave 1 of CHARLS in 2011, Wave 4 of ELSA in 2008–2009, and Wave 10 of HRS in 2010. Follow-up continued to Wave 5 of CHARLS in 2020, Wave 8 of ELSA in 2016–2017, and Wave 15 of HRS in 2020. Each cohort had received approval from its relevant ethics review authority, and written informed consent had been obtained from all participants.

The baseline years differed across cohorts. ELSA was assessed in 2008–2009, HRS in 2010, and CHARLS in 2011. This may have introduced differences in healthcare access, diagnostic practices, treatment availability, and population cardiometabolic trends. Because analyses were conducted separately within each cohort, these differences are unlikely to bias the within-cohort hazard ratio estimates. Still, effect sizes should be compared across cohorts with caution. Follow-up durations were broadly similar, at approximately 8–10 years.

**Figure 1:**
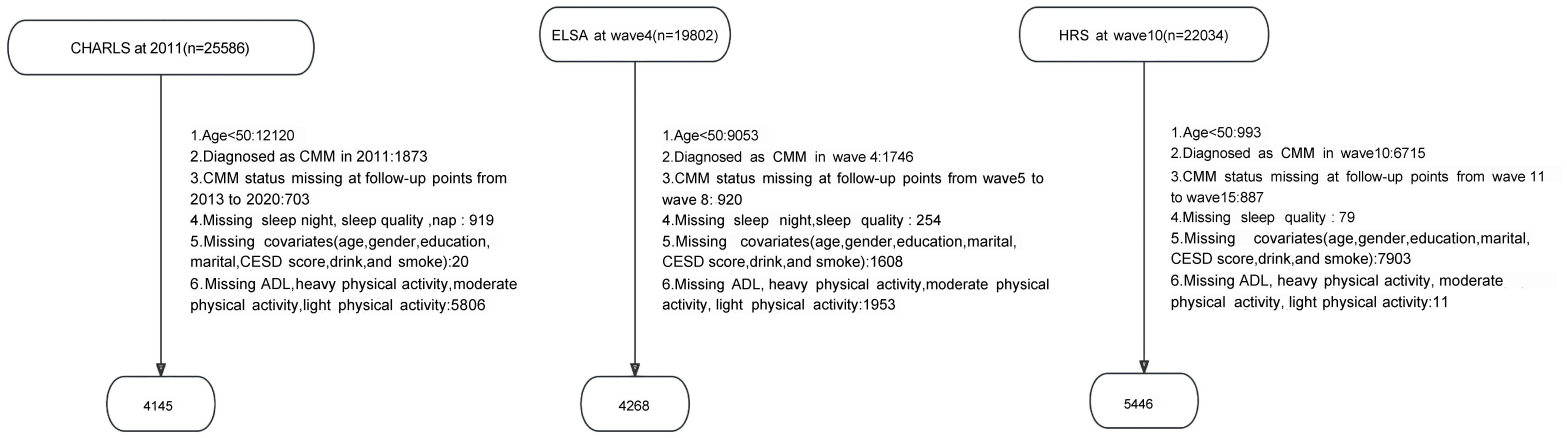
Selection process of the study population.

Figure 1 outlines participant selection. Across CHARLS, ELSA, and HRS, 67,422 individuals were screened at the outset. After excluding 22,166 participants younger than 50 years, 45,256 adults aged 50 years or older remained. Next, 10,334 participants diagnosed with CMM at baseline were excluded, leaving 34,922 participants. To examine the incident development of CMM, 2,510 participants whose CMM status was missing at follow-up were excluded because their outcome status could not be ascertained over the follow-up period. Excluding 1,252 participants who were missing sleep-related variables reduced the sample to 31,160. An additional 17,301 participants were excluded because of missing key variables required for analytic cohort construction. The remaining 13,859 participants made up the analytic cohort.

### 2.2 Sleep patterns assessment

Because the three cohorts used different instruments and response formats, nighttime sleep duration, daytime napping, and sleep quality were defined according to the original survey items and then recoded into analytically comparable categories for parallel analyses. In CHARLS, nighttime sleep duration was assessed using the self-reported question, “During the past month, how many hours of actual sleep did you get every night?”, and was categorized as short (≤6 h/night), normal (7–8 h/night), and long (≥9 h/night) (23). Daytime napping was assessed by self-reported post-lunch nap duration and, for the present analysis, classified as <30 min/day, 30–50 min/day, and >50 min/day. The intermediate category encompassed the previously reported optimal range of approximately 42–50 min/day among Chinese older adults (24). Sleep quality was derived from the reported frequency of sleeping poorly during the past week and categorized as good (<1 day/week), fair (1–4 days/week), and poor (5–7 days/week) (25). For ELSA, nighttime sleep duration was divided into short (≤6 h/night), normal (7–8 h/night), and long (≥9 h/night) categories; the original continuous sleep-duration measure was also retained for sensitivity analyses (26). Sleep quality in ELSA was classified according to the sleep complaint score as good (4–7 points), fair (8–11 points), and poor (12–16 points) (27). HRS measured sleep disturbance with a modified four-item Jenkins Sleep Scale that captured difficulty falling asleep, difficulty maintaining sleep, early morning awakening, and feeling rested on waking (28, 29). Item responses were coded on a three-point scale, with the final item reverse-scored before calculating the total score. Summed scores ranged from 4 to 12, with higher totals indicating greater sleep disturbance (29). To align the variable across cohorts, HRS sleep quality was classified as good (4–6), fair (7–9), or poor (10–12). Because the sleep questionnaires differed in design across cohorts, harmonization was guided by conceptual equivalence rather than identical raw coding, preserving the validity of the original measurements while improving cross-cohort comparability. Cross-cohort comparisons should therefore be interpreted at the construct level rather than as direct equivalences between raw scores.

The harmonization procedure followed four explicit steps. (1) Source items: the original sleep-related items were extracted from each cohort (single-item self-reports in CHARLS; continuous nighttime sleep hours and the multi-item sleep-complaint score in ELSA; the modified four-item Jenkins Sleep Scale in HRS). (2) Construct mapping: items were mapped to three target constructs—nighttime sleep duration (CHARLS and ELSA), sleep quality (all three cohorts), and daytime napping (CHARLS only, as ELSA and HRS did not include comparable items). Insomnia symptoms and chronotype were not separately analysable across cohorts. In HRS, insomnia-related symptom items were incorporated within the Jenkins Sleep Scale score and were therefore operationalized as part of the sleep-quality construct, whereas no comparable chronotype measure was available across cohorts. (3) Categorization: identical *a priori* cut-offs were used for nighttime sleep duration (≤6 h, 7–8 h, ≥9 h) across cohorts, while pre-specified cohort-specific thresholds based on the scoring structure of each instrument were used for sleep quality each yielding three ordinal categories (good / fair / poor). (4) Reporting caveat: because the original instruments differ, harmonization is at the construct level; cross-cohort comparisons should be interpreted as patterns of association rather than as direct equivalences between raw scores.

### 2.3 Definition of outcomes

Incident CMM was the outcome of interest. The same definition was used in CHARLS, ELSA, and HRS (30). CMM was defined as the co-occurrence of two or more of the following four conditions: hypertension, type 2 diabetes mellitus, coronary heart disease, and stroke.

### 2.4 Covariate assessment

The models were adjusted for age, sex, educational level, marital status, smoking, drinking, physical activity, chronic lung disease, depression, body mass index, waist circumference, systolic blood pressure, and activities of daily living. Variable definitions were made as consistent as possible across CHARLS, ELSA, and HRS before analysis. Education was grouped into below high school and high school or above. Marital status was classified as married or non-married. Smoking and drinking were both coded as no or yes. Chronic lung disease was identified from self-reported history. Physical activity was considered separately according to vigorous-, moderate-, and light-intensity activity.

### 2.5 Statistical analysis

Statistical analyses were conducted in R 4.5.2, with two-sided testing throughout, and *P* < 0.05 indicated statistical significance. Continuous variables were reported as mean ± standard deviation, and categorical variables as number and percentage. For group comparisons, analysis of variance or the Kruskal–Wallis test was used for continuous data, whereas the chi-square test or Fisher's exact test was used for categorical data. Kaplan–Meier methods were applied to characterize cumulative CMM incidence across categories of sleep quality, nighttime sleep duration, and daytime napping, and the log-rank test was used for group comparisons.

Associations of baseline sleep quality, nighttime sleep duration, and daytime napping with incident CMM were estimated with cohort-specific Cox proportional hazards models and are reported as hazard ratios with 95% confidence intervals. The unadjusted model was followed by a model including age, sex, education level, and marital status, and then by a fully adjusted model further including smoking status, alcohol consumption, physical activity, chronic lung disease, depression, body mass index, waist circumference, systolic blood pressure, and activities of daily living. Potential nonlinear patterns were examined with restricted cubic splines, and curves from both the crude and fully adjusted models were presented. Mediation analyses were conducted in the CHARLS and ELSA cohorts, using blood pressure, to explore potential pathways linking nighttime sleep duration to CMM. Diastolic blood pressure served as the candidate mediator, whereas systolic blood pressure was instead included as a covariate in the fully adjusted model; in addition, low-density lipoprotein cholesterol (LDL-C), high-density lipoprotein cholesterol (HDL-C), total cholesterol (TC), triglycerides, and C-reactive protein (CRP) were each examined as separate candidate mediators within the same analytic framework, to capture lipid- and inflammation-related pathways; direct and indirect effects were decomposed and reported with 95% confidence intervals. Participants with missing follow-up CMM status, missing primary sleep variables, or missing key variables required for construction of a unified analytic cohort were excluded. This unified cohort was used to maintain consistency across the primary Cox regression, mediation, and machine learning analyses. For the retained analytic samples, remaining missing values in selected covariates were imputed using chained equations. A single completed dataset, corresponding to the fifth imputed dataset, was used for the primary Cox regression analyses. Sensitivity analyses were conducted using the nonimputed analytic dataset, and the primary and sensitivity analyses are presented separately. Finally, we applied interpretable machine learning methods to assess the relative contribution of sleep variables within a broader cardiometabolic risk framework. The following R packages were used: “survival” and “survminer” for Cox proportional hazards models and Kaplan–Meier curves; “rms” for restricted cubic splines; “mediation” for mediation analyses with bootstrapped 95% confidence intervals; “mice” for chained-equation imputation of missing covariate values; the “mlr3” ecosystem (including “mlr3verse,” “mlr3tuning,” “mlr3learners,” and “mlr3extralearners”) for the machine-learning pipeline, including logistic regression (classif.log_reg), XGBoost, LightGBM, neural network, random forest, k-nearest neighbors, support vector machine, and naive Bayes; “pROC” and “mlr3measures” for performance evaluation; and “shapviz” and “SHAPforxgboost” for SHAP-based interpretation. Plots were produced using “ggplot2” and “patchwork.”

### 2.6 Model building

CHARLS was randomly divided into a training set and an internal test set at a 7:3 ratio, and ELSA was used for external validation. Because HRS did not include a comparable nighttime sleep duration variable, the machine learning analysis was performed using CHARLS for model development and ELSA for validation. The prediction model was constructed specifically to quantify the contribution of nighttime sleep duration to incident CMM, so external validation required a cohort in which this exposure had been measured in a comparable way. Although HRS was the second largest cohort (*n* = 5,446), it did not assess nighttime sleep duration with a comparable item and therefore could not validate a model that includes this variable; using HRS would have required removing the key sleep-duration feature and would not have provided a valid test of the model as specified. ELSA was the only cohort other than CHARLS that provided a comparable nighttime sleep duration measure, and was accordingly used for external validation. HRS was instead retained for the Cox proportional hazards and Kaplan–Meier analyses of sleep quality, for which a harmonized measure was available across all three cohorts.

Eight classifiers were compared, including logistic regression, XGBoost, LightGBM, neural network, random forest, k-nearest neighbors, support vector machine, and naive Bayes. Model performance was assessed using five-fold cross-validation, internal testing, and external validation. ROC curves and precision-recall curves were used to evaluate discrimination. Calibration curves and decision curve analysis were used to assess calibration and potential clinical utility. SHAP analysis was used to examine the relative contribution of predictors.

## 3 Results

### 3.1 Baseline characteristics

A total of 13,859 participants from three aging cohorts met the inclusion criteria and were included in the analysis. These included 4,145 participants from CHARLS, 4,268 from ELSA, and 5,446 from HRS. Baseline characteristics of the three cohorts are shown in Tables 1–3. The cohorts differed in several aspects. Educational attainment was lower in CHARLS than in ELSA and HRS. Smoking was more common in CHARLS, whereas alcohol consumption was more common in ELSA. For cardiometabolic indicators, participants in HRS had higher mean BMI, waist circumference, and diastolic blood pressure, while those in CHARLS had lower levels of these indicators.

**Table 1:** Baseline characteristics of participants in ELSA according to incident CMM status.

Variable	Overall *N* = 4268^a^	No incident CMM *N* = 3704^a^	Incident CMM *N* = 564^a^	*p-*Value^b^
AGE	64.12 (8.58)	63.56 (8.31)	67.77 (9.36)	<0.001
Gender				<0.001
Female	2,307 (54.1%)	2,041 (55.1%)	266 (47.2%)	
Male	1,961 (45.9%)	1,663 (44.9%)	298 (52.8%)	
Marital				0.010
Married	3,051 (71.5%)	2,674 (72.2%)	377 (66.8%)	
Non married	1,217 (28.5%)	1,030 (27.8%)	187 (33.2%)	
Education				0.024
Below high school	859 (20.1%)	766 (20.7%)	93 (16.5%)	
High school and above	3,409 (79.9%)	2,938 (79.3%)	471 (83.5%)	
Smoke				0.9
No	3,749 (87.8%)	3,255 (87.9%)	494 (87.6%)	
Yes	519 (12.2%)	449 (12.1%)	70 (12.4%)	
Drink				0.002
No	1,196 (28.0%)	1,007 (27.2%)	189 (33.5%)	
Yes	3,072 (72.0%)	2,697 (72.8%)	375 (66.5%)	
Chronic lung disease				<0.001
No	4,089 (95.8%)	3,568 (96.3%)	521 (92.4%)	
Yes	179 (4.2%)	136 (3.7%)	43 (7.6%)	
Sleep night	6.89 (1.21)	6.91 (1.19)	6.77 (1.34)	0.009
Sleep quality				0.076
Poor	819 (19.2%)	698 (18.8%)	121 (21.5%)	
Fair	1,789 (41.9%)	1,542 (41.6%)	247 (43.8%)	
Good	1,660 (38.9%)	1,464 (39.5%)	196 (34.8%)	
BMI	27.85 (4.97)	27.59 (4.86)	29.55 (5.32)	<0.001
Systolic blood pressure	131.96 (17.11)	130.86 (16.76)	139.17 (17.59)	<0.001
Diastolic blood pressure	75.26 (10.45)	75.00 (10.31)	76.97 (11.18)	<0.001
Depression	456 (10.7%)	382 (10.3%)	74 (13.1%)	0.053
Waist	95.88 (13.30)	95.06 (13.00)	101.26 (13.99)	<0.001
ADL				<0.001
No	3,752 (87.9%)	3,289 (88.8%)	463 (82.1%)	
Yes	516 (12.1%)	415 (11.2%)	101 (17.9%)	
Heavy activity				<0.001
Never	1,033 (24.2%)	946 (25.5%)	87 (15.4%)	
1 per week	940 (22.0%)	835 (22.5%)	105 (18.6%)	
>1 per week	2,295 (53.8%)	1,923 (51.9%)	372 (66.0%)	
Moderate activity				<0.001
Never	2,938 (68.8%)	2,604 (70.3%)	334 (59.2%)	
1 per week	880 (20.6%)	745 (20.1%)	135 (23.9%)	
>1 per week	450 (10.5%)	355 (9.6%)	95 (16.8%)	
Light activity				<0.001
Never	3,478 (81.5%)	3,051 (82.4%)	427 (75.7%)	
1 per week	527 (12.3%)	444 (12.0%)	83 (14.7%)	
>1 per week	263 (6.2%)	209 (5.6%)	54 (9.6%)	

a Mean (SD) or n (%).

b Two Sample t-test; Pearson's Chi-squared test.

**Table 2:** Baseline characteristics of participants in CHARLS according to incident CMM status.

Variable	Overall *N* = 4145^a^	No incident CMM *N* = 3326^a^	Incident CMM *N* = 819^a^	*p* Value^b^
Age	61.48 (8.04)	61.21 (8.11)	62.56 (7.66)	<0.001
Gender				0.001
Female	2,123 (51.2%)	1,661 (49.9%)	462 (56.4%)	
Male	2,022 (48.8%)	1,665 (50.1%)	357 (43.6%)	
Marital				0.2
Married	3,441 (83.0%)	2,774 (83.4%)	667 (81.4%)	
Non married	704 (17.0%)	552 (16.6%)	152 (18.6%)	
Education				0.8
Below high school	3,710 (89.5%)	2,974 (89.4%)	736 (89.9%)	
High school and above	435 (10.5%)	352 (10.6%)	83 (10.1%)	
Smoke				0.003
No	2,481 (59.9%)	1,953 (58.7%)	528 (64.5%)	
Yes	1,664 (40.1%)	1,373 (41.3%)	291 (35.5%)	
Drink				0.4
No	2,497 (60.2%)	1,992 (59.9%)	505 (61.7%)	
Yes	1,648 (39.8%)	1,334 (40.1%)	314 (38.3%)	
Chronic lung disease				0.019
No	3,719 (89.7%)	3,003 (90.3%)	716 (87.4%)	
Yes	426 (10.3%)	323 (9.7%)	103 (12.6%)	
Sleep night	6.34 (1.89)	6.34 (1.89)	6.33 (1.86)	0.8
Sleep quality				0.003
Poor	791 (19.1%)	609 (18.3%)	182 (22.2%)	
Fair	1,308 (31.6%)	1,034 (31.1%)	274 (33.5%)	
Good	2,046 (49.4%)	1,683 (50.6%)	363 (44.3%)	
Nap	30.83 (41.47)	29.75 (40.99)	35.21 (43.10)	<0.001
BMI	23.05 (3.51)	22.66 (3.35)	24.66 (3.66)	<0.001
Systolic blood pressure	129.81 (21.04)	127.68 (20.35)	138.49 (21.57)	<0.001
Diastolic blood pressure	74.90 (11.86)	74.04 (11.74)	78.39 (11.70)	<0.001
Depression	1,516 (36.6%)	1,178 (35.4%)	338 (41.3%)	0.002
Waist	83.98 (12.08)	82.93 (11.40)	88.25 (13.73)	<0.001
ADL				<0.001
No	3,463 (83.5%)	2,838 (85.3%)	625 (76.3%)	
Yes	682 (16.5%)	488 (14.7%)	194 (23.7%)	
Heavy activity				<0.001
Never	1,416 (34.2%)	1,195 (35.9%)	221 (27.0%)	
1 per week	50 (1.2%)	43 (1.3%)	7 (0.9%)	
>1 per week	2,679 (64.6%)	2,088 (62.8%)	591 (72.2%)	
Moderate activity				0.4
Never	2,323 (56.0%)	1,880 (56.5%)	443 (54.1%)	
1 per week	57 (1.4%)	46 (1.4%)	11 (1.3%)	
>1 per week	1,765 (42.6%)	1,400 (42.1%)	365 (44.6%)	
Light activity				0.5
Never	3,303 (79.7%)	2,661 (80.0%)	642 (78.4%)	
1 per week	40 (1.0%)	33 (1.0%)	7 (0.9%)	
>1 per week	802 (19.3%)	632 (19.0%)	170 (20.8%)	

a Mean (SD) or n (%).

b Two Sample t-test; Pearson's Chi-squared test.

**Table 3:** Baseline characteristics of participants in HRS according to incident CMM status.

Variable	Overall *N* = 5446^a^	No incident CMM *N* = 4059^a^	Incident CMM *N* = 1387^a^	*p* Value^b^
Age	64.04 (10.23)	63.44 (10.09)	65.78 (10.44)	<0.001
Gender				>0.9
Female	3,201 (58.8%)	2,388 (58.8%)	813 (58.6%)	
Male	2,245 (41.2%)	1,671 (41.2%)	574 (41.4%)	
Marital				0.020
Married	3,306 (60.7%)	2,501 (61.6%)	805 (58.0%)	
Non married	2,140 (39.3%)	1,558 (38.4%)	582 (42.0%)	
Education				0.001
Below high school	521 (9.6%)	357 (8.8%)	164 (11.8%)	
High school and above	4,925 (90.4%)	3,702 (91.2%)	1,223 (88.2%)	
Smoke				0.3
No	4,586 (84.2%)	3,432 (84.6%)	1,154 (83.2%)	
Yes	860 (15.8%)	627 (15.4%)	233 (16.8%)	
Drink				<0.001
No	2,033 (37.3%)	1,436 (35.4%)	597 (43.0%)	
Yes	3,413 (62.7%)	2,623 (64.6%)	790 (57.0%)	
Chronic lung disease				<0.001
No	5,120 (94.0%)	3,842 (94.7%)	1,278 (92.1%)	
Yes	326 (6.0%)	217 (5.3%)	109 (7.9%)	
Sleep quality				<0.001
Poor	586 (10.8%)	405 (10.0%)	181 (13.0%)	
Fair	3,035 (55.7%)	2,240 (55.2%)	795 (57.3%)	
Good	1,825 (33.5%)	1,414 (34.8%)	411 (29.6%)	
BMI	29.44 (6.09)	28.88 (5.81)	31.06 (6.58)	<0.001
Systolic blood pressure	129.23 (20.51)	127.30 (19.72)	134.89 (21.70)	<0.001
Diastolic blood pressure	80.47 (11.99)	79.75 (11.58)	82.60 (12.87)	<0.001
Depression	1,027 (18.9%)	709 (17.5%)	318 (22.9%)	<0.001
Waist	99.75 (15.14)	98.24 (14.79)	104.17 (15.32)	<0.001
ADL				<0.001
No	4,832 (88.7%)	3,665 (90.3%)	1,167 (84.1%)	
Yes	614 (11.3%)	394 (9.7%)	220 (15.9%)	
Heavy activity				<0.001
Never	1,631 (29.9%)	1,291 (31.8%)	340 (24.5%)	
1 per week	1,200 (22.0%)	911 (22.4%)	289 (20.8%)	
>1 per week	2,615 (48.0%)	1,857 (45.8%)	758 (54.7%)	
Moderate activity				<0.001
Never	2,969 (54.5%)	2,297 (56.6%)	672 (48.4%)	
1 per week	1,646 (30.2%)	1,204 (29.7%)	442 (31.9%)	
>1 per week	831 (15.3%)	558 (13.7%)	273 (19.7%)	
Light activity				0.002
Never	3,346 (61.4%)	2,548 (62.8%)	798 (57.5%)	
1 per week	1,772 (32.5%)	1,280 (31.5%)	492 (35.5%)	
>1 per week	328 (6.0%)	231 (5.7%)	97 (7.0%)	

a Mean (SD) or n (%).

b Two Sample t-test; Pearson's Chi-squared test.

### 3.2 Cox regression analyses

Among the sleep variables examined, sleep quality showed the most consistent association with CMM. In Model 3, good sleep quality was associated with a lower risk of CMM compared with poor sleep quality in CHARLS (HR = 0.79, 95% CI: 0.65–0.97, *P* = 0.023) and ELSA (HR = 0.76, 95% CI: 0.60–0.98, *P* = 0.035). A similar direction was observed in HRS, although the association did not reach statistical significance (HR = 0.84, 95% CI: 0.70–1.00, *P* = 0.056). Fair sleep quality was not significantly associated with CMM risk in any cohort (CHARLS: *P* = 0.823; HRS: *P* = 0.236; ELSA: *P* = 0.588).

After full adjustment, nighttime sleep duration was not significantly associated with CMM risk in CHARLS or ELSA. In CHARLS, neither sleep-duration category differed significantly from the reference group (HR = 0.89 and 0.94; *P* = 0.547 and 0.406). Similar nonsignificant results were observed in ELSA (HR = 0.79 and 0.85; *P* = 0.516 and 0.168). HRS did not include a comparable measure of nighttime sleep duration.

In ELSA, each 1-h increase in nighttime sleep duration was associated with a lower risk of CMM in Model 1 (HR = 0.92, *P* = 0.011) and Model 2 (HR = 0.93, *P* = 0.028), but the association was no longer significant in Model 3 (HR = 0.95, 95% CI: 0.89–1.02, *P* = 0.166). Daytime napping was assessed only in CHARLS and was not significantly associated with CMM risk after full adjustment, either for 30–50 min/day (HR = 1.13, 95% CI: 0.88–1.43, *P* = 0.340) or >50 min/day (HR = 1.11, 95% CI: 0.96–1.29, *P* = 0.158).

Overall, sleep quality showed the most consistent association with CMM across the three cohorts. The detailed Cox regression results are shown in Table 4, and a cross-cohort summary of the main sleep-related findings is provided in Table 5. Because sleep measures were harmonized at the construct level rather than by identical raw scores, comparisons across cohorts should focus on the overall pattern rather than on exact numerical differences in hazard ratios.

**Table 4:** Cox proportional hazards regression analyses.

Variable	Model 1		Model 2		Model 3	
	HR(95%CI)	*p*-Value	HR(95%CI)	*p*-Value	HR(95%CI)	*p*-Value
CHARLS						
Sleep quality						
Poor	Ref.		Ref.		Ref.	
Fair	0.9(0.7441–1.0825)	0.258	0.92(0.7593–1.1049)	0.359	0.98(0.8068–1.186)	0.823
Good	0.72(0.6049–0.8635)	<0.001	0.76(0.6309–0.9038)	0.002	0.79(0.6503–0.9693)	0.023
Sleep night						
≤6 h	Ref.		Ref.		Ref.	
7–8 h	0.9(0.6313–1.291)	0.575	0.89(0.6198–1.2691)	0.511	0.89(0.6226–1.2854)	0.547
≥9 h	0.87(0.7516–1.0128)	0.073	0.92(0.7923–1.0708)	0.285	0.94(0.7982–1.0955)	0.406
Nap						
<30 min	Ref.		Ref.		Ref.	
30–50 min	1.19(0.9374–1.5192)	0.151	1.2(0.9436–1.531)	0.136	1.13(0.8829–1.4345)	0.34
>50 min	1.27(1.1006–1.4729)	0.001	1.3(1.122–1.5087)	<0.001	1.11(0.959–1.2935)	0.158
ELSA						
Sleep quality						
Poor	Ref.		Ref.		Ref.	
Fair	0.9(0.7281–1.1248)	0.368	0.82(0.6586–1.0223)	0.078	0.94(0.7441–1.1824)	0.588
Good	0.76(0.608–0.9566)	0.019	0.68(0.5414–0.8624)	0.001	0.76(0.596–0.9809)	0.035
Sleep night						
≤6 h	Ref.		Ref.		Ref.	
7–8 h	1.08(0.5249–2.2237)	0.834	0.88(0.4263–1.8131)	0.727	0.79(0.3784–1.6296)	0.516
≥9 h	0.71(0.5678–0.8848)	0.002	0.76(0.6076–0.9505)	0.016	0.85(0.6717–1.072)	0.168
Sleep night	0.92(0.8566–0.9806)	0.011	0.93(0.8682–0.9919)	0.028	0.95(0.8936–1.0195)	0.166
HRS						
Sleep quality						
Poor	Ref.		Ref.		Ref.	
Fair	0.81(0.691–0.9543)	0.011	0.86(0.729–1.0087)	0.064	0.9(0.767–1.0675)	0.236
Good	0.67(0.5665–0.8037)	<0.001	0.76(0.6376–0.9095)	0.003	0.84(0.6964–1.0046)	0.056

HR, hazard ratio; CI, confidence interval; CMM, cardiometabolic multimorbidity. Model 1 was unadjusted. Model 2 was adjusted for age, sex, educational level, and marital status. Model 3 was adjusted for age, sex, marital status, education level, smoking, alcohol drinking, physical activity, chronic lung disease, depression, body mass index (BMI), waist circumference, systolic blood pressure, and activities of daily living (ADL).

**Table 5:** Summary of the main associations between sleep patterns and incident cardiometabolic multimorbidity across three cohorts.

Sleep dimension	Comparison	CHARLS HR (95% CI); *p* value	ELSA HR (95% CI); *p* value	HRS HR (95% CI); *p* value	Overall interpretation
Sleep quality	Fair vs poor	0.98 (0.8068–1.1860); *P* = 0.823	0.94 (0.7441–1.1824); *P* = 0.588	0.90 (0.7670–1.0675); *P* = 0.236	No significant association after full adjustment.
Sleep quality	Good vs poor	0.79 (0.6503–0.9693); *P* = 0.023	0.76 (0.5960–0.9809); *P* = 0.035	0.84 (0.6964–1.0046); *P* = 0.056	Protective direction across cohorts; significant in CHARLS and ELSA and borderline in HRS.
Nighttime sleep duration	7–8 h vs ≤ 6 h	0.89 (0.6226–1.2854); *P* = 0.547	0.79 (0.3784–1.6296); *P* = 0.516	Not available	No significant association after full adjustment.
Nighttime sleep duration	≥9 h vs ≤ 6 h	0.94 (0.7982–1.0955); *P* = 0.406	0.85 (0.6717–1.0720); *P* = 0.168	Not available	No significant association after full adjustment.
Daytime napping	>50 min vs < 30 min	1.11 (0.9590–1.2935); *P* = 0.158	Not available	Not available	Available only in CHARLS; not significant after full adjustment.
Daytime napping	30–50 min vs < 30 min	1.13 (0.8829–1.4345); *P* = 0.340	Not available	Not available	Available only in CHARLS; not significant after full adjustment.

HR, hazard ratio; CI, confidence interval; CMM, cardiometabolic multimorbidity. CMM was defined as the co-occurrence of two or more of hypertension, type 2 diabetes mellitus, coronary heart disease, and stroke. Results are from the fully adjusted Cox proportional hazards model (Model 3). Model 3 was adjusted for age, sex, marital status, education level, smoking, alcohol drinking, physical activity (vigorous, moderate, and light), chronic lung disease, depression, body mass index (BMI), waist circumference, systolic blood pressure, and activities of daily living (ADL). Not available indicates that the corresponding sleep variable was not measured in a comparable way in that cohort. Sleep variables were harmonized at the construct level; therefore, cross-cohort comparisons should be interpreted as patterns of association rather than direct equivalences between raw scores.

### 3.3 KM survival curves

**Figure 2:**
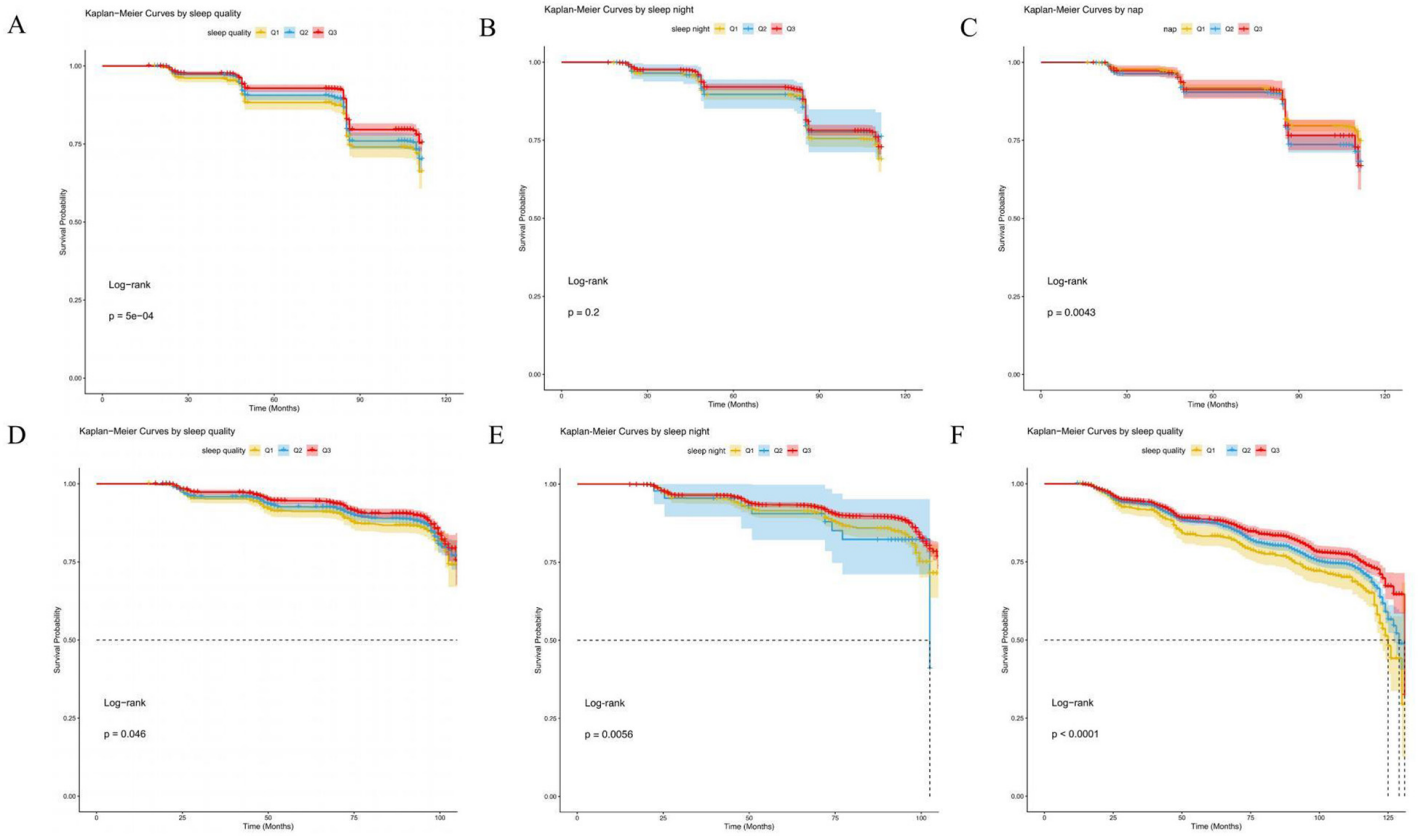
Kaplan–Meier analyses of survival free from incident cardiometabolic multimorbidity according to sleep-related variables. **(A–C)** show results from CHARLS: **(A)** sleep quality, **(B)** nighttime sleep duration, and **(C)** daytime napping. **(D, E)** show results from ELSA: **(D)** sleep quality and **(E)** nighttime sleep duration. **(F)** shows results from HRS: **(F)** sleep quality. For sleep quality, Q1, Q2, and Q3 indicate poor, fair, and good sleep quality, respectively. For nighttime sleep duration in CHARLS and ELSA, Q1, Q2, and Q3 indicate ≤ 6 h/night, 7–8 h/night, and ≥9 h/night, respectively. For daytime napping in CHARLS, Q1, Q2, and Q3 indicate < 30 min/day, 30–50 min/day, and >50 min/day, respectively. *P*-values were calculated using the log-rank test.

As shown in Figure 2, the probability of remaining free of CMM differed across several sleep-related categories. In CHARLS, significant differences were observed for sleep quality (*P* = 0.0005) and daytime napping (*P* = 0.0043), whereas nighttime sleep duration showed no significant difference (*P* = 0.20). In ELSA, the curves differed by sleep quality (*P* = 0.046) and nighttime sleep duration (*P* = 0.0056). In HRS, sleep quality was also associated with a significant difference between curves (*P* < 0.0001). Overall, the KM results were generally consistent with the Cox models, with sleep quality showing the most stable association across cohorts.

### 3.4 Restricted cubic spline analyses

**Figure 3:**
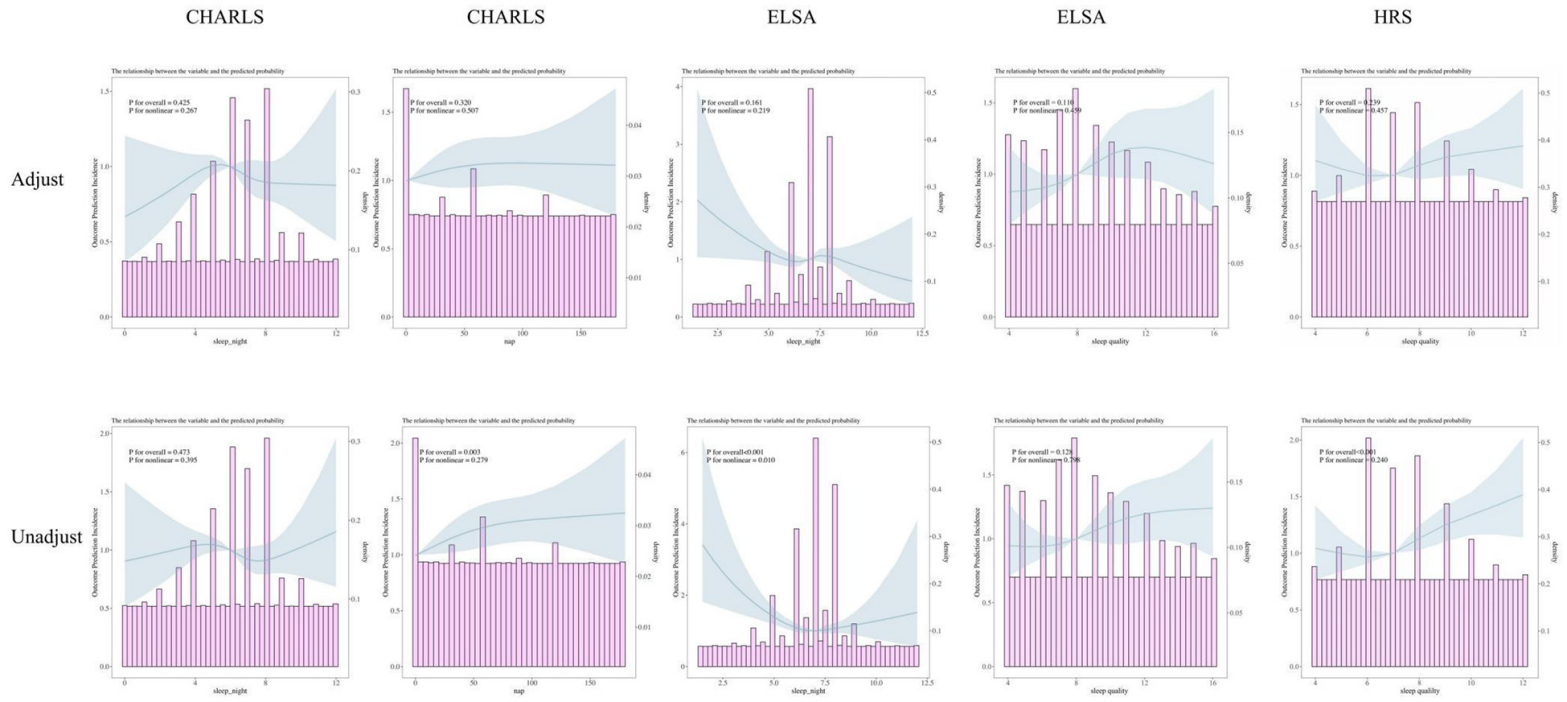
Restricted cubic spline analyses of the associations between continuous sleep measures and incident CMM. Curves were estimated using Cox proportional hazards models; therefore, the vertical scale should be interpreted as relative hazard estimates rather than absolute predicted probabilities. Adjust and Unadjust indicate the fully adjusted and crude models, respectively. The adjusted models included age, sex, marital status, education level, smoking, alcohol drinking, physical activity, chronic lung disease, depression, body mass index, waist circumference, systolic blood pressure, and activities of daily living.

Figure 3 presents the cohort-specific associations between continuous sleep measures and incident CMM estimated using Cox spline models. In ELSA and HRS, summed sleep-quality scores were modeled as continuous variables and interpreted within each cohort. Overall, no consistent nonlinear association remained after multivariable adjustment. In ELSA, nighttime sleep duration showed a U-shaped pattern in the crude model, with the lowest hazard around 7–8 h and higher hazards at shorter and longer durations (*P* for nonlinearity = 0.010), but this pattern was attenuated after adjustment. In HRS, higher sleep-quality scores, indicating poorer sleep quality, were associated with a higher hazard before adjustment, whereas the association was no longer evident after adjustment. In CHARLS, the crude association between daytime napping and incident CMM was also attenuated after adjustment, and nighttime sleep duration showed no clear association.

### 3.5 Mediation analysis

**Figure 4:**
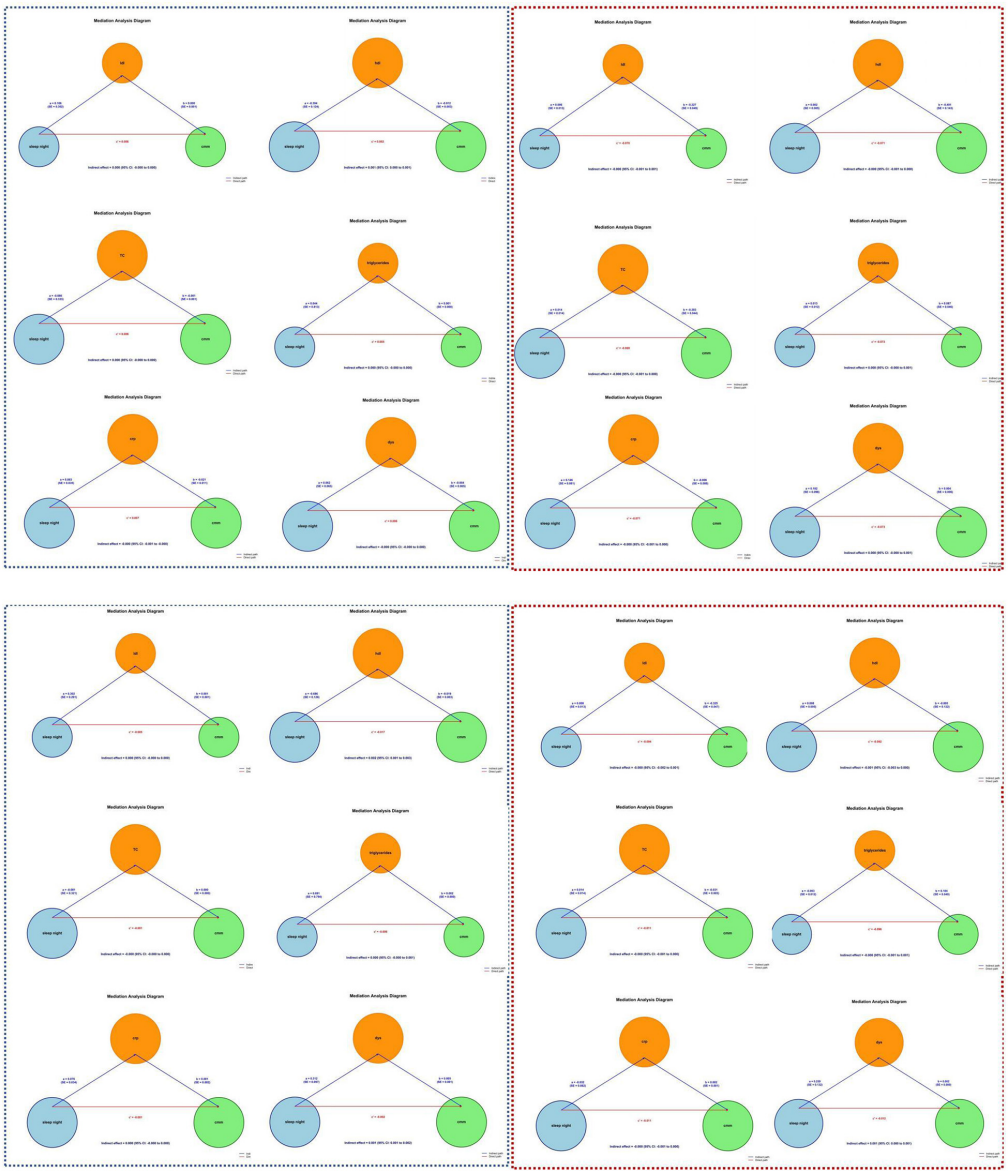
Exploratory mediation analyses of the association between nighttime sleep duration and incident CMM in CHARLS and ELSA. DBP, LDL-C, HDL-C, TC, triglycerides, and CRP were examined separately as candidate mediators. Blue and red dashed boxes indicate CHARLS and ELSA, respectively; the upper and lower panels show covariate-adjusted and crude analyses, respectively. Indirect effects and 95% confidence intervals were estimated using 5,000 bootstrap resamples. Interpretation focused on the adjusted analyses.

Figure 4 shows the mediation analyses for nighttime sleep duration and incident CMM in CHARLS and ELSA. DBP, LDL-C, HDL-C, TC, triglycerides, and CRP were examined as potential mediators. Systolic blood pressure was included as a covariate rather than as a mediator. Both crude and covariate-adjusted models were assessed.

Some small indirect effects were observed in the crude analyses, but none of the examined mediators showed a clear indirect effect after covariate adjustment. These findings should therefore be interpreted as exploratory.

### 3.6 Sensitivity analysis

Table 6 presents sensitivity analyses based on the nonimputed analytic dataset. The directions of the main associations were broadly similar to those in the primary analyses, although some differences in statistical significance were observed. In HRS, the association between good sleep quality and lower CMM risk reached statistical significance in the nonimputed analysis.

**Table 6:** Sensitivity analysis.

Variable	Model 1		Model 2		Model 3	
	HR (95%CI)	*p*-Value	HR (95%CI)	*p*-Value	HR (95%CI)	*p*-Value
CHARLS						
Sleep quality						
Poor	Ref.		Ref.		Ref.	
Fair	0.91(0.7382–1.1129)	0.348	0.92(0.7471–1.1267)	0.411	0.97(0.7881–1.201)	0.798
Good	0.72(0.5908–0.8727)	<0.001	0.74(0.6094–0.9032)	0.003	0.8(0.639–0.9911)	0.041
Sleep night						
≤6 h	Ref.		Ref.		Ref.	
7–8 h	0.81(0.5342–1.2228)	0.314	0.8(0.5255–1.2051)	0.281	0.83(0.548–1.2682)	0.395
≥9 h	0.87(0.7382–1.0218)	0.089	0.91(0.772–1.0719)	0.258	0.94(0.7906–1.1175)	0.483
Nap						
<30 min	Ref.		Ref.		Ref.	
30–50 min	1.24(0.9583–1.6158)	0.101	1.25(0.9598–1.6207)	0.098	1.18(0.904–1.5288)	0.227
>50 min	1.3(1.1066–1.5215)	0.001	1.32(1.1263–1.5567)	<0.001	1.14(0.9696–1.3435)	0.112
ELSA						
Sleep quality						
Poor	Ref.		Ref.		Ref.	
Fair	0.83(0.6867–1.0058)	0.057	0.74(0.6121–0.9009)	0.003	0.9(0.7308–1.1011)	0.299
Good	0.72(0.59–0.8767)	0.001	0.63(0.5103–0.7665)	<0.001	0.76(0.6087–0.9459)	0.014
Sleep night						
≤6 h	Ref.		Ref.		Ref.	
7–8 h	1.41(0.8101–2.4524)	0.225	1.15(0.6578–1.9987)	0.629	1.04(0.5976–1.8248)	0.879
≥9 h	0.71(0.5807–0.8617)	<0.001	0.73(0.599–0.8924)	0.002	0.86(0.6961–1.0521)	0.139
Sleep night	0.93(0.8756–0.9881)	0.019	0.93(0.8789–0.9903)	0.022	0.98(0.9203–1.0339)	0.402
HRS						
Sleep quality						
Poor	Ref.		Ref.		Ref.	
Fair	0.81(0.6842–0.9558)	0.013	0.86(0.7282–1.0193)	0.082	0.93(0.778–1.1161)	0.443
Good	0.66(0.5494–0.7902)	<0.001	0.75(0.6217–0.8995)	0.002	0.81(0.6642–0.99)	0.04

HR, hazard ratio; CI, confidence interval. Results are presented as HR (95%CI) and P values from Cox regression sensitivity analyses.

Model 1: unadjusted model.

Model 2: adjusted for age, sex, education level, and marital status.

Model 3: adjusted for age, sex, marital status, education level, smoking, alcohol drinking, physical activity (vigorous, moderate, and light), chronic lung disease, depression, body mass index (BMI), waist circumference, systolic blood pressure, and activities of daily living (ADL).

### 3.7 Machine-learning model performance and interpretation

**Figure 5:**
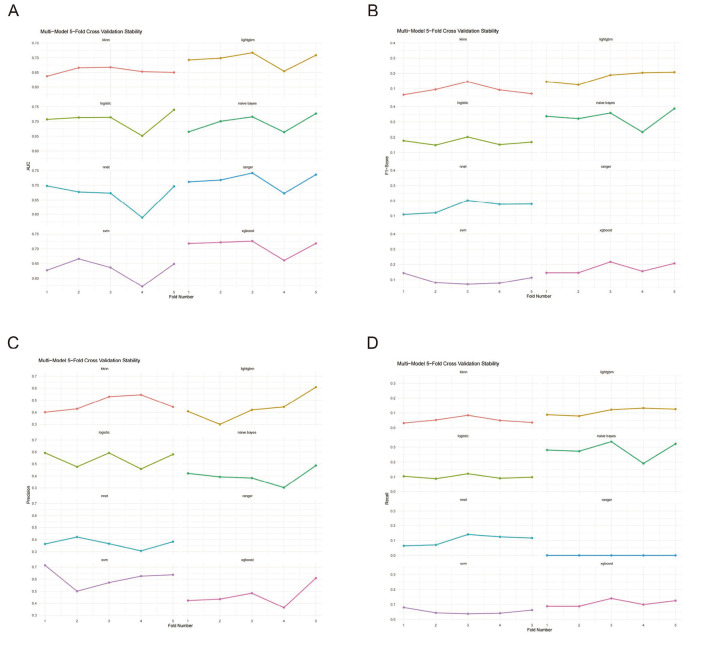
Five-fold cross-validation stability of the eight predictive models in the CHARLS training set. **(A)** shows the area under the receiver operating characteristic curve (AUC), **(B)** shows the F1 score, **(C)** shows precision, and **(D)** shows recall across the five cross-validation folds.

**Figure 6:**
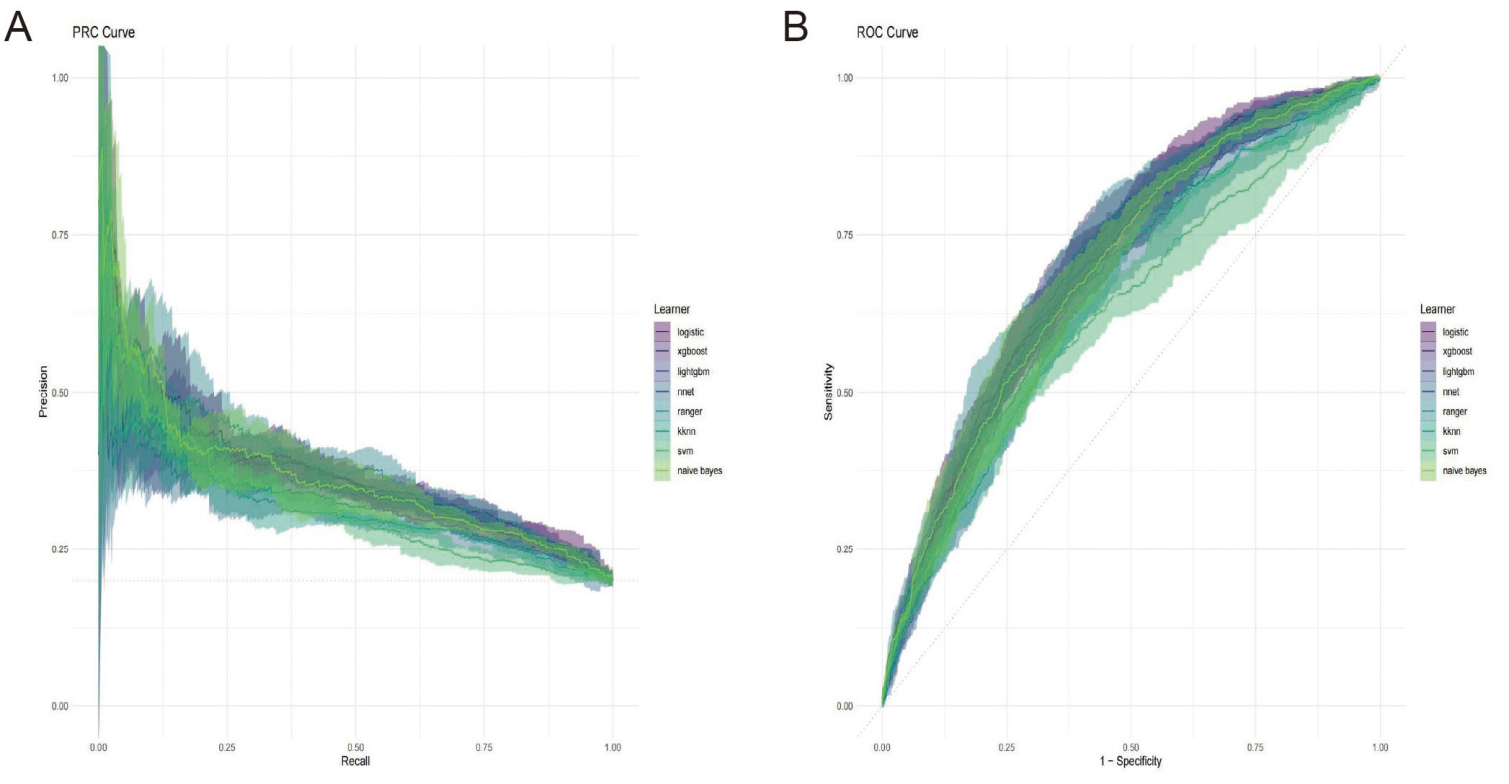
Five-fold cross-validated discrimination performance of the eight predictive models in the CHARLS training set. **(A)** shows the precision-recall curves, and **(B)** shows the receiver operating characteristic curves.

**Figure 7:**
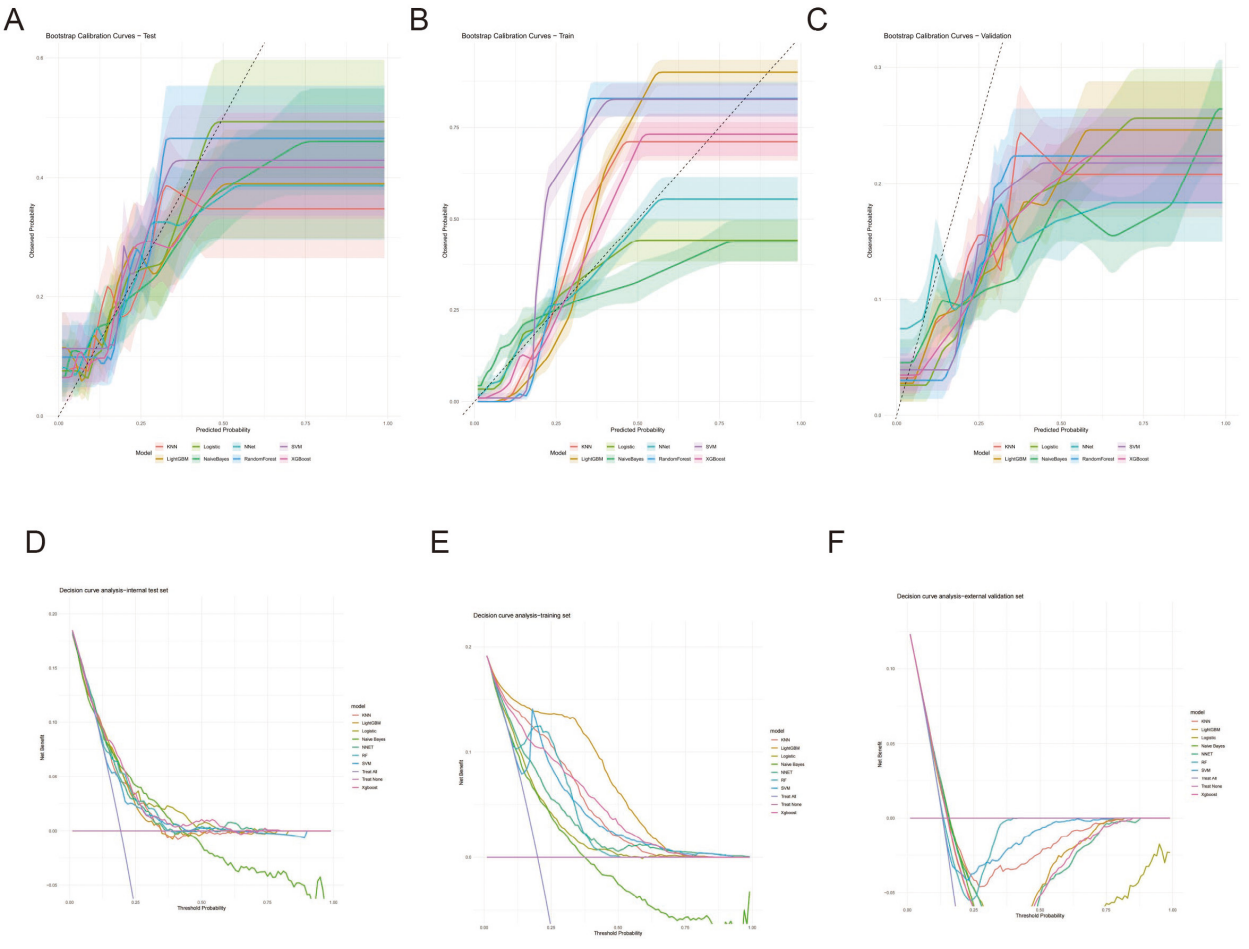
Calibration and decision curve analyses of the eight predictive models in the CHARLS model-development dataset and the ELSA external-validation dataset. **(A)** shows bootstrap calibration curves for the CHARLS internal test set, **(B)** shows bootstrap calibration curves for the CHARLS training set, and **(C)** shows bootstrap calibration curves for the ELSA external validation set. **(D)** shows decision curve analysis for the CHARLS internal test set, **(E)** shows decision curve analysis for the CHARLS training set, and **(F)** shows decision curve analysis for the ELSA external validation set. The shaded areas in **(A–C)** represent 95% bootstrap confidence intervals.

The five-fold cross-validation results showed broadly stable model performance, although the overall predictive ability was moderate (Figure 5). ROC and precision-recall curves also suggested modest discrimination (Figure 6). Calibration and decision curve analyses showed variable performance in the CHARLS training and internal test sets and weaker performance in the ELSA external validation set (Figure 7).

**Figure 8:**
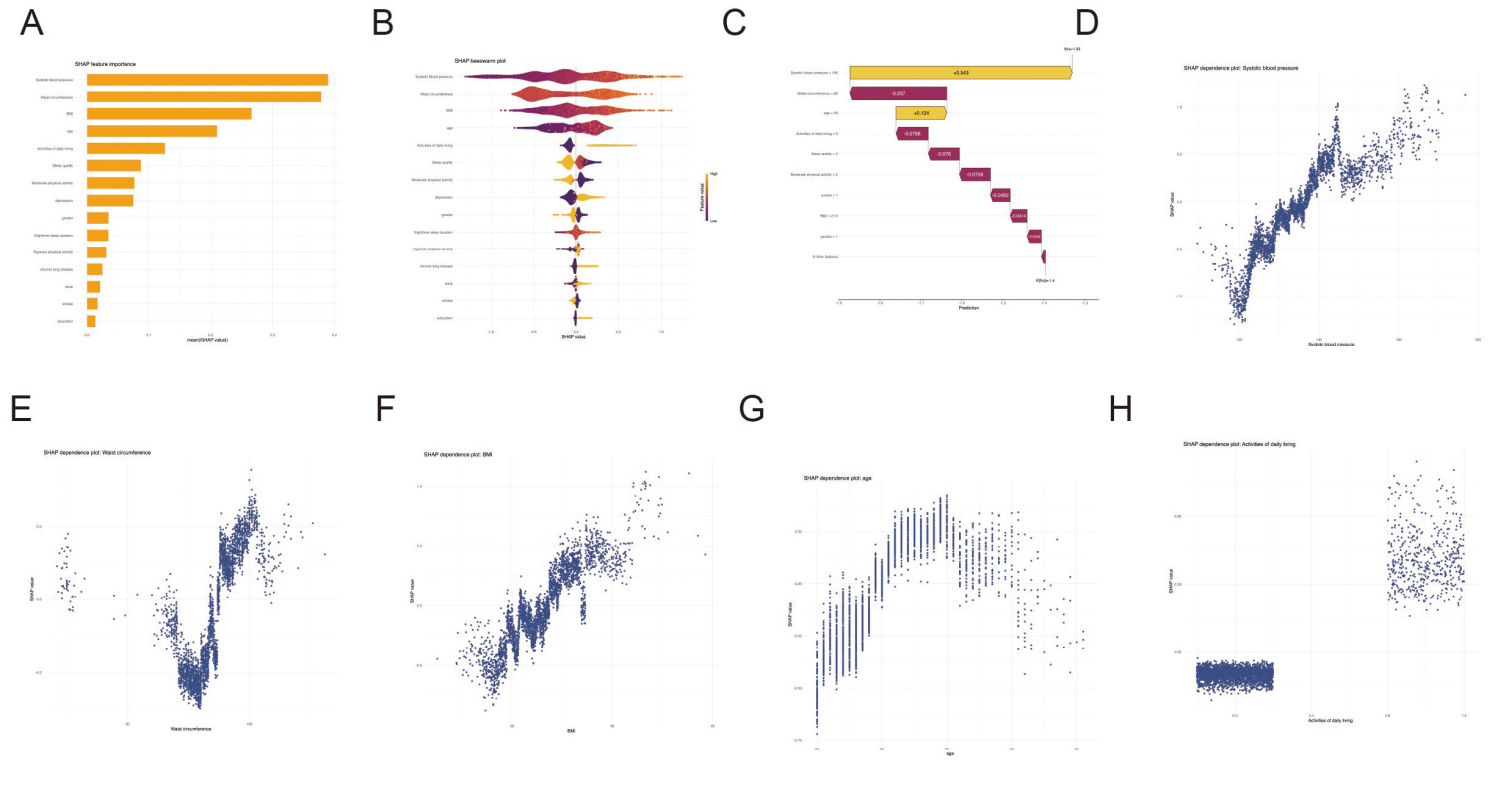
SHAP-based interpretation of the XGBoost model developed using the CHARLS training set. **(A)** shows global feature importance ranked according to the mean absolute SHAP values. **(B)** shows the SHAP beeswarm plot, with colors indicating the relative feature values. **(C)** shows a SHAP waterfall plot for an individual prediction. **(D–H)** show SHAP dependence plots for systolic blood pressure, waist circumference, body mass index, age, and activities of daily living, respectively.

In the SHAP analysis, systolic blood pressure, waist circumference, and body mass index were among the leading predictors of incident CMM. Age, activities of daily living, sleep quality, and nighttime sleep duration also contributed to prediction, but their relative importance was smaller (Figures 8A, B). The waterfall plot illustrated one individual prediction, whereas the dependence plots showed how systolic blood pressure, waist circumference, body mass index, age, and activities of daily living were associated with model output across observations (Figures 8C–H).

Overall, the machine learning findings were consistent with the traditional regression analyses. Blood pressure and anthropometric indicators contributed most strongly to CMM prediction, while sleep-related variables showed smaller but still observable contributions.

## 4 Discussion

We used data from three nationally representative aging cohorts in China, the United States, and the United Kingdom to examine whether nighttime sleep duration, sleep quality, and daytime napping were associated with incident cardiometabolic multimorbidity (CMM). Daytime napping was available only in CHARLS. The main finding was that good sleep quality was associated with a lower risk of incident CMM in CHARLS and ELSA. A similar direction was observed in HRS, although the association did not reach statistical significance. In contrast, nighttime sleep duration and daytime napping showed less consistent associations, and these associations were weakened after full adjustment. These results suggest that sleep quality may be a more stable sleep-related marker of CMM risk in older adults.

From a metabolic standpoint, poor sleep quality likely reflects a broader state of physiological dysregulation than what sleep duration alone can capture (8, 31). Beyond its association with unfavorable behavioral patterns, impaired sleep quality has been linked to neuroendocrine activation (32), autonomic imbalance (33), chronic low-grade inflammation (34), adverse blood pressure regulation (35), and adiposity-driven metabolic dysfunction (36), all of which may contribute to the long-term accumulation of cardiometabolic disease burden. These mechanisms may partly explain why sleep quality showed a more consistent association with incident CMM than other sleep variables in this study. Fair sleep quality was not significantly associated with CMM risk in any cohort, suggesting that the difference may be more evident between poor and good sleep quality rather than across adjacent categories. The findings for nighttime sleep duration were less consistent. In CHARLS and ELSA, categorical analyses suggested some differences across sleep-duration groups, but these associations were not clear after full adjustment. In ELSA, the continuous sleep-duration measure also lost significance after adjustment. This pattern suggests that the association between sleep duration and CMM may be influenced by covariates and may not be well described as a simple linear relationship. Our findings are broadly consistent with previous dose-response meta-analyses reporting U- or J-shaped associations between sleep duration and cardiometabolic outcomes, although the attenuation after multivariable adjustment indicates that confounding may partly account for these patterns. This pattern accords with the Whitehall II cohort, in which short sleep (≤5 h) at ages 50, 60, and 70 years was associated with a 30–40% higher risk of incident multimorbidity relative to 7-h sleep (HR 1.30–1.40) (37). The association between sleep quality and CMM observed in this study is broadly consistent with previous prospective studies linking poor sleep quality with cardiovascular disease, type 2 diabetes, and metabolic syndrome. For example, a UK Biobank study reported that hypertensive adults with the healthiest sleep pattern had a 27% lower risk of incident CMM than those with the least healthy sleep pattern (38). This estimate is similar in magnitude to the lower CMM risk observed for good vs. poor sleep quality in our analyses. In CHARLS, daytime napping was associated with a higher risk of CMM in the unadjusted analysis, but this association was no longer significant after full adjustment. This may partly reflect differences in baseline health, nighttime sleep disruption, or other unmeasured factors. Sensitivity analyses based on the nonimputed dataset showed broadly similar directions of association, although some differences in statistical significance were observed.

The exploratory mediation analyses did not provide clear evidence that DBP, lipid markers, or CRP explained the association after covariate adjustment. Other plausible mechanisms, such as chronic inflammation, circadian disruption, autonomic dysregulation, and metabolic imbalance, may also contribute (11, 13, 33).

The machine learning analyses provided a complementary view of CMM risk prediction. Overall model performance was moderate, and no model showed clear superiority across all evaluation steps. Calibration and decision curve analyses also suggested that the models should be interpreted cautiously, particularly in external validation. Therefore, the machine learning component is better viewed as an exploratory tool for risk stratification and feature interpretation rather than as a clinical screening model. Recent interpretable machine learning work has also been applied to CMM prediction in higher-risk clinical populations, such as patients with type 2 diabetes (39). SHAP analysis identified systolic blood pressure, waist circumference, and BMI as the leading contributors to CMM prediction. Sleep-related variables, including nighttime sleep duration and sleep quality, also contributed to model prediction, but their relative importance was smaller. This pattern is consistent with the epidemiological analyses, in which traditional cardiometabolic risk factors showed stronger predictive roles than sleep variables. Rather than positioning sleep as a dominant standalone predictor, these results point to its role as one element within a broader metabolic and vascular risk architecture that underpins CMM.

These findings may have practical implications, but they should be interpreted cautiously because of the observational design. Sleep quality assessment may be useful in cardiometabolic risk evaluation for middle-aged and older adults. Our results suggest that good sleep quality, rather than small differences between adjacent sleep-quality categories, may be more relevant to CMM risk. The findings for nighttime sleep duration also support the view that both short and long sleep may be unfavorable, rather than longer sleep always being better (8). The mediation findings were exploratory and did not identify a clear explanatory pathway after covariate adjustment.

Several limitations should be considered. First, sleep variables were self-reported in all three cohorts, which may have introduced recall bias and measurement error. Future studies using actigraphy or polysomnography could provide more accurate sleep assessment. Second, CMM was identified from self-reported physician diagnoses. This approach may miss undiagnosed conditions, especially early hypertension and type 2 diabetes. Combining self-reported diagnoses with clinical examination or biomarker data would improve outcomeascertainment.

The mediation analyses were also limited by the available variables. We examined blood pressure, lipid markers, and CRP, but other relevant pathways could not be fully assessed. These include insulin resistance, cytokine-related inflammation, circadian regulation, autonomic function, and changes in biomarkers over time. Therefore, the biological mechanisms linking sleep to CMM remain only partly explained in this study.

The machine learning models should also be interpreted with caution. Although the models were useful for comparing predictor importance, their overall predictive performance was moderate. They should therefore be viewed as exploratory tools rather than clinical screening models. In addition, the prediction model was externally validated only in ELSA. HRS was not used for external validation because it did not include a nighttime sleep duration variable comparable to that in CHARLS and ELSA. Therefore, the generalizability of the model still needs to be tested in other cohorts with similar sleep-duration measures.

Residual confounding remains possible. We adjusted for physical activity, including vigorous-, moderate-, and light-intensity activity, but harmonized information on diet, obstructive sleep apnea, and detailed medication use was not available across all cohorts. The lack of dietary data is particularly relevant because diet may be related to both sleep and cardiometabolic risk. Future studies should include validated dietary assessments, such as food-frequency questionnaires or 24-h dietary recalls, together with objective sleep measures.

Another limitation is that sleep was measured only at baseline in all three cohorts. Sleep habits may change during follow-up, and these changes may be important for the development of CMM. For example, worsening or improving sleep quality, or changes in nighttime sleep duration over time, may carry different risks from a single baseline sleep measure. Because repeated sleep data were not available in the present analysis, we could not assess long-term sleep trajectories or separate participants with stable sleep from those whose sleep changed over time. This may have weakened the observed associations, especially for nighttime sleep duration. Therefore, our findings should be interpreted as associations between baseline sleep status and later CMM, rather than as evidence on the effects of long-term sleep patterns. Future studies with repeated sleep assessments are needed to examine how changes in sleep over time relate to CMM risk, including trajectory-based analyses such as group-based trajectory modeling. Finally, differences in measurement tools, follow-up protocols, and baseline survey years may have contributed to heterogeneity across CHARLS, ELSA, and HRS. Sleep variables were harmonized at the construct level rather than by identical raw scores, so effect sizes should not be compared as direct numerical equivalents across cohorts. Baseline data were collected in different calendar periods, including ELSA in 2008–2009, HRS in 2010, and CHARLS in 2011. These differences may reflect changes in healthcare access, diagnostic practice, and population characteristics. For this reason, cross-cohort findings should be interpreted mainly in terms of overall patterns rather than exact effect-size comparisons. Relatedly, sleep quality was categorized as good, fair, or poor for the primary Cox analyses. In the restricted cubic spline analyses, the original summed scores were used in ELSA and HRS, whereas sleep quality in CHARLS remained a categorical variable. Because ELSA and HRS used different instruments, the spline results were interpreted within each cohort rather than compared directly across cohorts. The three-level classification was retained to improve comparability and ensure stable estimates.

## 5 Conclusions

Among older adults from China, the United Kingdom, and the United States, better sleep quality was associated with a lower risk of cardiometabolic multimorbidity. This association was statistically significant in CHARLS and ELSA, and showed a similar direction in HRS, although it did not reach statistical significance. In contrast, nighttime sleep duration showed less consistent associations across cohorts with available data, with evidence of a nonlinear pattern in ELSA before full adjustment. These findings suggest that sleep quality may be a more stable sleep-related marker of CMM risk than sleep duration in older adults. The machine learning analyses identified blood pressure and anthropometric indicators as the main contributors to CMM prediction, while sleep-related variables showed smaller contributions.

## Data Availability

The datasets analyzed in this study are available from the CHARLS, ELSA, and HRS data repositories upon registration or application to the respective cohort data providers. Further inquiries can be directed to the corresponding author.
